# Water Quality and Microbial Community in the Context of Ecological Restoration: A Case Study of the Yongding River, Beijing, China

**DOI:** 10.3390/ijerph192013056

**Published:** 2022-10-11

**Authors:** Jie Li, Yujiao Sun, Xiaoyue Zhang, Chengzhong Pan, Shurong Zhang, Binghui Zheng

**Affiliations:** 1 College of Water Sciences, Beijing Normal University, Beijing 100875, China; 2 National Engineering Laboratory for Lake Pollution Control and Ecological Restoration, Chinese Research Academy of Environmental Sciences, Beijing 100012, China; 3 State Key Laboratory of Earth Surface Processes and Resource Ecology, Faculty of Geographical Science, Beijing Normal University, Beijing 100875, China

**Keywords:** ecological restoration, water quality, microbial community, Yongding River

## Abstract

Ecological water replenishment via interbasin water diversion projects provides opportunities for ecological river restoration. Untangling water quality changes, microbiota dynamics, and community functions is necessary for sustainable ecological management. Using the Yongding River as a case study, we monitored the water quality and applied genomic sequencing to investigate microbial communities of the river in different stages after ecological water replenishment. Our results showed that river water quality represented by chemical oxygen demand (COD), total nitrogen (TN), and chlorophyll-a (Chl-a) did not change significantly during months after water replenishment. The bacterial community composition varied in different months and river subsections. The *Cyanobium*_PCC-6307, CL500-29 marine group, and *Pseudomonas* were dominant in the later stages after water replenishment. Water temperature, pH, and nutrient levels significantly affected the microbial community composition, and ecological restoration may have the potential to influence nitrogen cycling in the river. Our results can provide ecological insights into sustainable water quality maintenance and river management following ecological restoration enabled by ecological water replenishment.

## 1. Introduction

Overexploitation and utilization of water resources, land use changes in river basins, and anthropogenic pollution have caused deterioration in river ecological systems [1,2,3]. The rise in river degradation globally has spurred research on river ecological restoration [4,5]. Water shortages in some areas can be effectively addressed by large-scale water transfer projects, such as the Chinese South-to-North Water Diversion Projects, the largest interbasin water transfer projects in the world [6,7]. As a measure of river ecological restoration, ecological water replenishment enabled by interbasin water transfer projects may optimize the allocation of water resources, restore the connectivity of rivers and lakes, and repair damaged river ecosystems [8,9]. Following the restoration of river water levels and volumes in water-scarce areas, the focus has shifted to the aquatic environmental changes caused by restoration projects [10]. However, the water quality and microecological status in rivers replenished by ecological water, which is river water intended for ecological restoration purposes, remain unclear.

Effective river restoration requires a complete understanding of the whole river ecological system, including the physical, chemical, and biological parameters [11,12]. Water quality may be changed by habitat restoration, and microbes are closely associated with variations in specific environmental factors, such as chemical oxygen demand, total nitrogen, and nitrate nitrogen [13,14]. Because microorganisms play a vital role in river health, increased attention has been given to microbial diversity and related ecosystem processes [15]. Numerous studies have suggested that microbial diversity and community composition may provide important biological indications for cyanobacterial proliferation in estuarine reservoirs [16]. In addition, microorganisms may help to develop an index of biotic integrity for the assessment of the ecological status of freshwater rivers [17] and aid in the development of a bacterial community-based index for the ecological status assessment of estuarine and coastal environments [18]. A robust understanding of the mechanisms of microbial community formation and succession is a prerequisite for indicating and predicting ecological environment status [19,20].

Since 2019, the Yongding River in Beijing has received ecological water replenishment through an interbasin water diversion project, which is formally known in China as the Yellow River Diversion Project [21]. The physicochemical and biological characteristics of the river after water replenishment remain unclear. In this study, changes in water quality and spatiotemporal dynamics of the microbial community along the Yongding River were investigated after water replenishment. This study aimed to answer the following key questions: (i) Did the water quality change significantly during the months after water replenishment? (ii)How did the spatiotemporal patterns of microbial community differ in river ecosystem? (iii) Which physicochemical properties were correlated with changes in microbial community structure? After obtaining results from genomic sequencing and statistical analysis, we examined how the water quality and microbial communities responded to the ecological restoration process based on ecological water replenishment.

## 2. Materials and Methods

### 2.1. Site Description and Sample Collection

The Yongding River has received ecological water replenishment through the Yellow River Diversion Project, and the Guanting Reservoir is a key water body within this interbasin project [21,22]. In our study, the Guanting Reservoir was regarded as the starting point in the Beijing section of the Yongding River (Figure S1). Water replenishment for the Beijing section of the Yongding River mainly depended on the discharge flow from the Guanting Reservoir; the peak flow in 2019 occurred in May and late September (Figure S2). Therefore, we defined the period from June to September as the post water replenishment period. June was defined as the early stage. August and September were defined as the later stages after water replenishment. We investigated both physicochemical water qualities and waterborne microbial communities during the months following water replenishment.

The Beijing section of the Yongding River was divided into two subsections. The upstream subsection of the river is located in a mountainous area, which is slightly disturbed by human activities. The predominant land use type in this area is forest. The downstream subsection of the river is located in an urban area, which is more disturbed by human activities. The predominant land use type here is impervious surface. Sites 1–5 are located in the upstream subsection, and sites 6–8 are located in the downstream subsection (Figure S1). A total of 24 surface water samples were collected along the Yongding River from eight sites in June, August, and September (Figure S2). Water samples were collected in sterile bottles and transported to the laboratory at 4 °C within 4 h for subsequent analysis [23].

### 2.2. Water Chemistry and Parameter Measurements

Water quality parameters, including temperature (T), pH, electrical conductivity (EC), oxidation–reduction potential (ORP), turbidity (Tur), and dissolved oxygen (DO), were detected using a HQ40d portable multiparameter instrument (HACH, USA). Chemical oxygen demand (COD) was measured by rapid digestion spectrophotometry, and ammonia nitrogen was detected according to Nessler’s reagent spectrophotometry. Total nitrogen (TN), total phosphorus (TP), and chlorophyll-a (Chl-a) were measured according to the standard methods established by the State Environmental Protection Administration in China [24].

### 2.3. DNA Extraction, Sequencing, and Analysis

Water samples were filtered through 0.2 µm filters (47 mm diameter, Millipore, Billerica, MA, USA) within 24 h after collection and the membranes were stored at −80 °C for DNA extraction. Total DNA extraction from each sample was performed according to a modified protocol using cetyltrimethylammonium bromide (CTAB) [25]. DNA concentration and purity were determined with a NanoDrop 2000 UV–vis spectrophotometer (Thermo Scientific, Wilmington, NC, USA). DNA samples were stored at −80 °C until subsequent amplification. The V3–V4 region of the 16S rRNA gene was amplified by using the primer pair 338F (5′-ACT CCT ACG GGA GGC AGC A-3′) and 806R (5′-GGA CTA CHV GGG TWT CTA AT-3′). Gene amplification was conducted; the PCR parameters were 95 °C for 3 min, followed by 30 cycles of 95 °C for 30 s, 50 °C for 30 s, and 72 °C for 45 s, with a final extension at 72 °C for 10 min. PCR reactions were performed in triplicate. Purified amplicons were pooled in equimolar amounts and paired-end sequenced on an Illumina MiSeq PE300 platform (Illumina, San Diego, USA). According to the quality-control process, low-quality reads were discarded, and high-quality sequencing data were retained. The taxonomy of sequences was identified using RDP Classifier (http://rdp.cme.msu.edu, accessed on 29 April 2022) according to the SILVA (Release 138) reference database (http://www.arb-silva.de, accessed on 29 April 2022). Alpha diversity indices, including richness and the Ace and Chao1 indices, which were used to calculate the community richness, and the Shannon index, which represented the community diversity, were analyzed using Mothur (version v.1.30.2) [26].

### 2.4. Statistical Analysis

Differences in the physicochemical factors of different months (June, August, and September) and different river sections (upstream and downstream sections) were compared by the nonparametric Kruskal–Wallis H test and Mann–Whitney U test using SPSS Statistics v26 (IBM Corp., Armonk, NY, USA). A *p* value below 0.05 was considered to be indicative of a statistically significant difference. Principal coordinate analysis (PCoA) was implemented based on Bray–Curtis distances to investigate differences between microbial communities across river sections and sampling months. Analysis of similarities (ANOSIM) was used to evaluate the significant differences (*p*) and the degree of separation among the above groups. The metabolic and ecologically relevant functions of microbes were predicted by functional annotation of prokaryotic taxa (FAPROTAX) [27]. The severity of multicollinearity among environmental factors was determined using variance inflation factor (VIF) analysis, and the environmental factors with VIF > 20 were removed. Then, canonical correspondence analysis (CCA) was conducted to explore the important factors influencing bacterial communities and reveal relationships between environmental factors and microbial communities [28].

## 3. Results

### 3.1. Spatiotemporal Comparison of Environmental Factors

Physicochemical properties were measured for all samples (Table 1). Water temperature (T) ranged between 20.75 and 32.20 °C during the sampling period, and pH ranged from 8.50 to 9.07, with a mean of 8.82. The dissolved oxygen (DO) concentrations ranged from 7.71 to 19.69 mg/L. Chlorophyll-a (Chl-a) concentrations ranged from 1.27 to 90.66 mg/L, indicating the different eutrophication conditions.

Water temperature, pH, oxidation–reduction potential (ORP), DO, and total phosphorus (TP) showed significant differences for different months (Table 1). The water temperature was significantly higher in August than in June (*p* < 0.001) and September (*p* < 0.05). The ORP was significantly higher in August than in September (*p* < 0.001). The DO was significantly higher in August than in June (*p* < 0.05). The TP was also significantly higher in August than in June (*p* < 0.01). Water quality represented by chemical oxygen demand (COD), total nitrogen (TN), nitrate nitrogen (NO_3_^−^-N), ammonia nitrogen (NH_4_^+^-N), and Chl-a showed no significant difference during the months after water replenishment.

The mean values of COD (*p* < 0.05), TP (*p* < 0.05), and Chl-a (*p* < 0.001) were significantly higher in the downstream samples than in the upstream samples (Table S1). The concentrations of total nitrogen and nitrate nitrogen showed higher mean values upstream, with nonsignificant differences. The water temperature, pH, turbidity, and DO showed no significant differences between the upstream and downstream sections.

### 3.2. Spatiotemporal Comparison of Microbial Communities

#### 3.2.1. Comparison of Alpha Diversity

A total of 24 water samples generated 1,302,388 optimized sequences, which were clustered into 3262 operational taxonomic units (OTUs). The alpha diversity of the bacterial communities in all samples in the different groups is shown in Table 2. The richness ranged from 409 to 1229 (Table S2). The Shannon index indicated a diversity range across all samples from 2.83 to 4.71. The Ace index ranged from 684.7 to 2167.0, and Chao1 ranged between 592.8 and 1781.2. Overall, microbial alpha diversity did not show significant changes over time (*p* > 0.05). The Ace and Chao1 indices of the upstream water were higher than those of the downstream water, but the differences were not statistically significant (*p* > 0.05).

#### 3.2.2. Composition of Bacterial Communities

**Figure 1:**
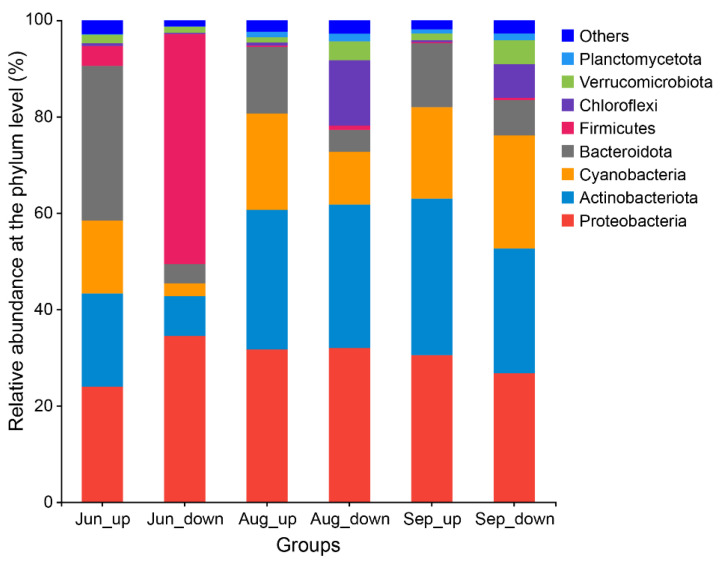
Microbial community composition at the phylum level. Jun, Aug, and Sep represent the sample collection months June, August, and September, respectively. Up and down indicate the sampling sites in the upstream and downstream subsections, respectively.

The bacterioplankton abundance is shown in Figure 1. Bacterial communities in August were structurally similar to the communities in September; the predominant phyla were Proteobacteria, Actinobacteriota, and Cyanobacteria. However, the composition of the bacterial community in these two months differed widely from that in June, which was dominated by Proteobacteria (27.90%), Bacteroidota (21.55%), and Firmicutes (20.52%).

In terms of the spatial effect, the dominant phyla were Proteobacteria (28.72%), Actinobacteriota (26.95%), Bacteroidota (19.72%), and Cyanobacteria (18.02%) in the upstream water. Proteobacteria (31.07%), Actinobacteriota (21.32%), Firmicutes (16.37%), and Cyanobacteria (12.34%) possessed the highest relative abundance in the downstream water. During the same period, the microbial community composition also differed in the upstream and downstream sections. The dominant communities of the river’s upper sections in June were Bacteroidota (32.10%) and Proteobacteria (23.96%), while the lower sections were dominated by Firmicutes (47.81%) and Proteobacteria (34.48%).

The heatmap and hierarchical clustering tree at the genus level display clear clusters based on the monthly differences (Figure S3). One cluster represents the samples from August and September and the upstream samples from June, and the other represents the downstream samples from June. The actinobacterial hgcI clade was relatively abundant in all samples. The dominant genera varied in different stages after ecological water replenishment. *Cyanobium*_PCC-6307, the CL500-29 marine group, and *Pseudomonas* were dominant in August and September. *Exiguobacterium*, *Acinetobacter*, and *Planococcus* were predominant in the downstream water in June.

**Figure 2:**
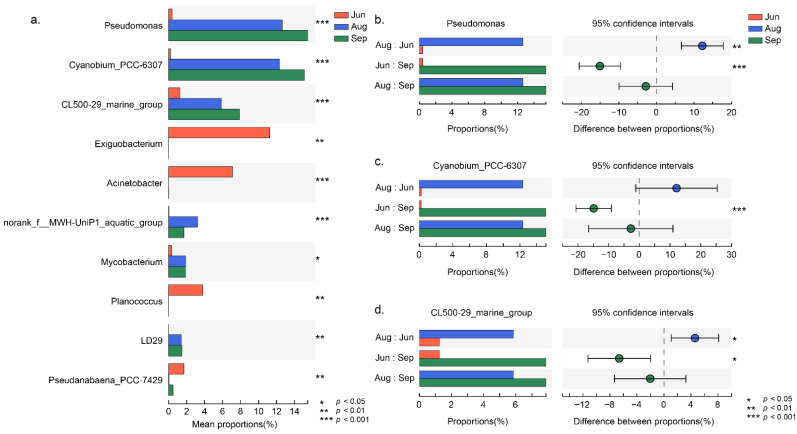
The difference in microbial community composition in different months at the genus level. Bar plots showing differences in (**a**) genera with high relative abundance, (**b**) *Pseudomonas*, (**c**) *Cyanobium*_PCC-6307, and (**d**) CL500-29 marine group. Jun, Aug, and Sep represent the sample collection months June, August, and September, respectively.

The difference in microbial community composition at the genus level is shown in Figure 2; the Kruskal–Wallis H test was used to determine the differences in different months (Figure 2a). *Pseudomonas* and the CL500-29 marine group showed significant differences between June and the other two months (Figure 2b,d). *Cyanobium*_PCC-6307 was significantly higher in September than in June (*p* < 0.001) (Figure 2c). Unclassified *Chloroplast* showed no significant difference in the groups of different months but showed significantly higher relevance upstream than downstream (*p* < 0.05) (Figure S4).

#### 3.2.3. Comparison of Beta Diversity

**Figure 3:**
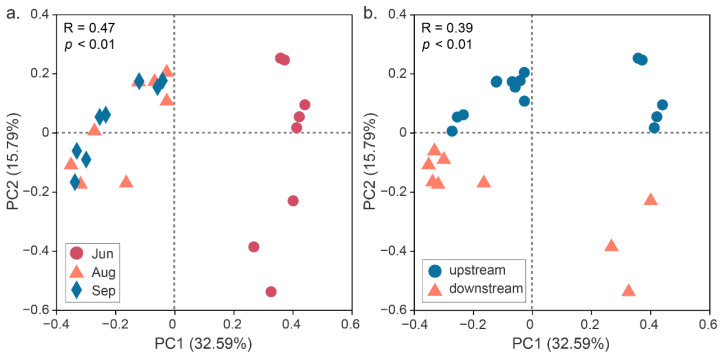
Principal coordinate analysis (PCoA) based on Bray–Curtis distances showing the distinct distribution patterns of samples in (**a**) June, August, and September, and (**b**) upstream and downstream sections. Analysis of similarities (ANOSIM) was used to test the significance of variations in community composition.

The difference in microbial community composition was arrayed by principal coordinate analysis (PCoA) based on Bray–Curtis distances (Figure 3). The first two principal components explained a total of 48.38% of the microbial community variance. Clustering by sampling period (Figure 3a) and by sampling subsections (Figure 3b) was observed. All samples in August and September were clustered together but distinct from those in June. The overall distribution pattern of bacterial community composition in the upstream samples differed significantly from that in the downstream samples (*p* < 0.01).

### 3.3. Functional Prediction for Microbial Communities

**Figure 4:**
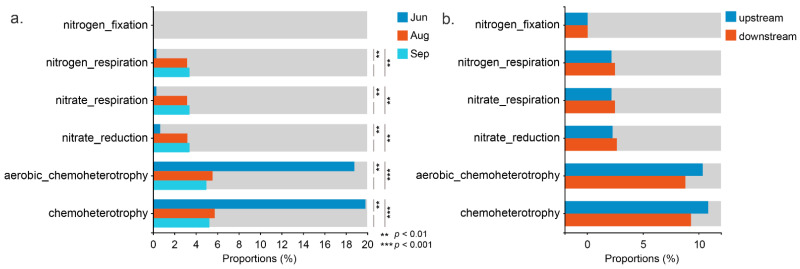
Functional annotation of prokaryotic taxa (FAPROTAX) analysis revealing the different functions in (**a**) June, August, and September, and (**b**) upstream and downstream sections.

The functional profiles of waterborne bacterial communities were predicted using the functional annotation of prokaryotic taxa (FAPROTAX). Forty functional profiles were annotated, and the functions we paid more attention to during the process of river restoration are shown in Figure 4. The two most abundant functional traits in all samples were chemoheterotrophy and aerobic chemoheterotrophy. The relative abundances of these two functions in the early stage after water replenishment were significantly higher than those in the later stages (*p* < 0.01). The proportion of bacterial functions related to nitrogen transformation, such as nitrogen respiration, nitrate respiration, and nitrate reduction, was significantly higher in August and September than in June (*p* < 0.01). Nitrogen fixation showed no significant difference in different months and sections, indicating a similar nitrogen fixation potential along the river in different stages after water replenishment.

### 3.4. Multivariate Analyses of Biotic and Abiotic Components

**Figure 5:**
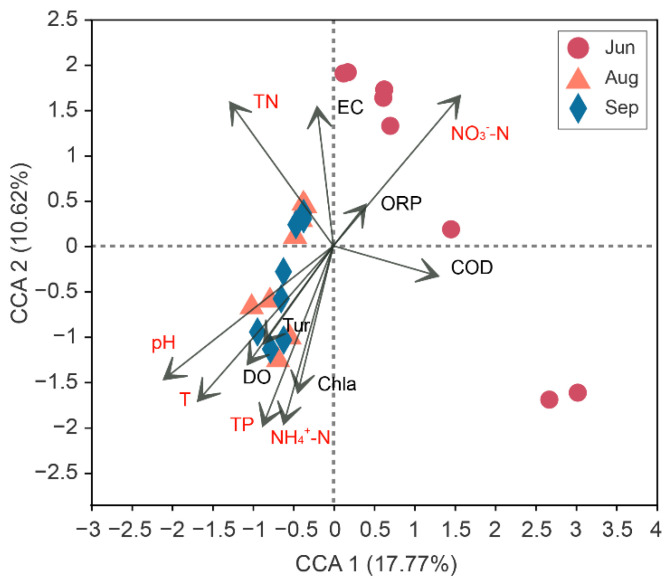
Canonical correspondence analysis (CCA) plots showing the relationship between environmental factors and microbial communities. Factors identified as significantly influencing bacterial communities are highlighted in red. Each point represents a single sample (*n* = 24). Jun, Aug, and Sep represent the sample collection months June, August, and September, respectively. T, water temperature; ORP, oxidation–reduction potential; EC, electrical conductivity; DO, dissolved oxygen; Tur, turbidity; COD, chemical oxygen demand; TN, total nitrogen; TP, total phosphorus; NH_4_^+^-N, ammonia nitrogen; NO_3_^−^-N, nitrate nitrogen; Chl-a, chlorophyll-a.

Canonical correspondence analysis (CCA) was used to show the correlation between environmental factors and microbial communities (Figure 5). Among all these environmental factors, water temperature (T), pH, TN, TP, NH_4_^+^-N, and NO_3_^−^-N were identified as exerting significant impacts on bacterial community composition (Table S3). Different forms of nitrogen had different effects on the samples. The samples in June were affected by NO_3_^−^-N, and the samples in August and September were influenced by NH_4_^+^-N (*p* < 0.05). Samples from August and September were clustered together and were affected by T, pH, and TP. In general, environmental factors played important roles in affecting the distribution of the microbial community in different months after water replenishment.

## 4. Discussion

### 4.1. Water Quality Status after Ecological Restoration in the Yongding River

The concentrations of COD, TP, and Chl-a were significantly higher in the downstream samples than in the upstream samples (Table S1). Two factors could account for the higher COD and TP concentrations in the downstream samples. First, the riparian buffer generated at low water levels was inundated extensively owing to the rise in river water level caused by water replenishment [29]. During the inundation process, dissolved organic matter and phosphorus from the previous riparian zone were re-released into the water column, which may have led to an increase in COD and TP. Second, the higher COD and TP concentrations downstream were also affected by the urban land use types, population density, and anthropogenic stressors, which were suggested to negatively influence river ecosystem health and lead to higher contaminant concentrations [28,30]. Furthermore, the high content of organic matter and phosphorus provided conditions for algal growth, resulting in significantly higher Chl-a content (*p* < 0.001) in the downstream samples compared to upstream samples [31,32].

There were no significant differences in COD, TN, or Chl-a between months (Table 1), indicating that no significant changes in these indices occurred during the four months after water replenishment. This result seemingly implied that the source water transported by ecological water replenishment was conducive to maintaining a relatively clean river water state for several months after replenishment. However, the concentrations of TP in August were significantly higher than those in June (*p* < 0.01). Meanwhile, water temperature was significantly higher in August than in the other two months (*p* < 0.05) and positively correlated with TP (*p* < 0.01) (Table S4). Given that the high TP concentration coincided with the high temperature, water quality monitoring should be strengthened, and management measures need to be taken seriously during periods of high temperatures after water replenishment [33].

### 4.2. Spatiotemporal Variations in Microbial Communities and Functions

The microbial diversity and richness did not show any significant difference between the early (June) and later (August and September) stages after ecological water replenishment. In contrast, the microbial community composition varied in different months (Figure 3a). The bacterial community composition in the water was similar in August and September, implying that the microbial community structure tends to be stable in the later stages after water replenishment. This result is in line with the general law of primary succession, which states that the taxonomic composition and functional profiles of communities become less variable with time in late successional stages [34]. In general, the characteristic differences in microbes with time were mainly reflected in the changes in bacterial community composition rather than bacterial alpha diversity (Figure 3, Table 2).

The results showed that water in the upstream section of the Yongding River supported similar richness to water in the downstream section (Table 2). However, the microbial community structures in these two sections were distinct (Figure 3b). The Yongding River watershed has been divided into the water conservation zone, plain urban zone, and plain suburban zone [21]. Our sampling sites in the upstream section are located in the water conservation zone, which is slightly disturbed by human activities. Sites in the downstream section are located in the plain urban zone, which is more disturbed by human activities. Intense human activities around rivers have been reported to greatly influence water quality during the rainy season because of runoff pollution [35] and may result in microbial changes. In addition to the ecological water replenishment, reclaimed water utilization was another strategy for ecological water allocation, and reclaimed water was utilized in the downstream sections. Despite the difference in water quality between reclaimed water and surface water [36], microorganisms that can survive in reclaimed water are structurally different from those adapted to surface water [37], possibly resulting in microbial community differences between the downstream and upstream river sections. We have discussed the factors that may affect upstream and downstream microorganisms, but it is unclear how these factors affect microorganism distribution. The mechanism by which these factors influence microbial community structure should be investigated further.

In the later stages after water replenishment, the relative abundance of *Pseudomonas*, *Cyanobium*_PCC-6307, and the CL500-29 marine group increased significantly. Members of *Pseudomonas*, belonging to Proteobacteria, are involved in functions related to plant health and plant growth [38,39]. It was reported that the *Cyanobium*_PCC-6307 and CL500-29 marine groups decreased with water flow in the interbasin water diversion canal of the South-to-North Water Diversion Projects and were restrained in growth close to the canal end [40], which suggests a negative effect of the water replenishment process on these two genera. We hypothesized that the replenishment process was the main factor causing the low abundance of these genera in the early stage after water replenishment. However, these microorganisms gradually recovered, increased, and occupied a high relative abundance in August and September, the later stages after water replenishment.

The functional prediction based on FAPROTAX analysis suggested that river restoration might change bacterial functions over time (Figure 4). Chemoheterotrophy and aerobic chemoheterotrophy were the major functions in June. The functions related to nitrogen transformation, such as nitrogen respiration, nitrate respiration, and nitrate reduction, showed higher relative abundance in the later stages after replenishment. The results implied that ecological restoration had the potential to influence element cycling, especially nitrogen cycling in the river, and promote the water autopurification ability [41]. It is worth noting that this functional prediction is biased in existing reference genomes and cannot directly reflect all functions of all microorganisms. Metagenomics and transcriptomics may be used to acquire accurate information about the function of microbial communities influenced by river restoration [42].

### 4.3. The Ecological Combination of Water Quality and Microorganisms

The composition of the microbial community upstream and downstream over time is affected by water quality. However, the effects of changes in water quality after ecological replenishment on the microbial ecosystems of rivers remain unclear [43]. Water temperature significantly influenced the microbial communities in August and September (*p* < 0.01) (Table S3). It has been reported that temperature greatly affects the bacterial community composition and significantly promotes the proliferation of *Synechococcus* in the warm season [16]. Monitoring water quality and ecology during the high-temperature season is particularly important. The nutrient parameters, including TN (R^2^ = 0.293, *p* < 0.05), TP (R^2^ = 0.347, *p* < 0.05), NH_4_^+^-N (R^2^ = 0.308, *p* < 0.05) and NO_3_^−^-N (R^2^ = 0.397, *p* < 0.01) (Table S3), also significantly affected the microbial community composition in the river. Nitrate had an impact on the water samples in the early stage, while ammonium mainly affected the samples in the later stages after water replenishment (Figure 5). Nitrogen and phosphorus are important factors that we should pay attention to during the process of ecological restoration of rivers.

In general, environmental factors representing the status of contaminants, such as COD, TN, and NH_4_^+^-N, showed no significant differences in the different months after water replenishment, which implies that ecological water replenishment enables the water quality of the Yongding River to stay relatively clean for a minimum of four months. Even so, monitoring the water quality of rivers impacted by human disturbance is still important throughout the water replenishment period. The microbial community composition varied in different stages and eventually tended to stabilize. It is difficult to demonstrate how this transition was affected or whether it was beneficial (e.g., enhancing water autopurification) or detrimental (e.g., promoting eutrophication). The extent to which our findings can be generalized to more ecologically replenished rivers is unclear. The role of microorganisms in the process of river restoration requires an in-depth explanation, and more attention should be given to the indicative role of microbes in the evaluation of river ecological restoration effects in the future.

## 5. Conclusions

Ecological restoration is an important international topic. The present study uncovered the water quality variations and underlying microbial community dynamics in an ecologically restored river. After ecological water replenishment, changes in water quality and microbial community composition in different stages were observed. Our results also implied a strong interplay between temporal, spatial, and environmental factors influencing microbial communities and functions in the river. Microbial communities are influenced by environmental changes, such as temperature and nutrient variations. Further research on the transition of microbial function may provide a shortcut for untangling the relationship between environmental factors and microorganisms, thus providing administrators and policy-makers with effective monitoring and management tools.

## Figures and Tables

**Table 1:** Comparison of environmental parameters in June, August, and September in the Beijing Section of the Yongding River.

Variables *	June		August		September	
	Mean	s.d.	Mean	s.d.	Mean	s.d.
T (°C)	22.35 ^b^	1.38	29.80 ^a^	1.73	24.60 ^b^	2.22
pH	8.69 ^b^	0.09	8.94 ^a^	0.10	8.84 ^ab^	0.10
ORP (mV)	116.55 ^ab^	12.54	139.85 ^a^	17.24	84.89 ^b^	30.42
EC (µS/cm)	1397.16 ^a^	584.08	1275.13 ^a^	141.55	1071.88 ^a^	69.66
DO (mg/L)	8.55 ^b^	0.70	11.14 ^a^	3.44	9.96 ^ab^	1.22
Tur (NTU)	5.66 ^a^	3.07	9.97 ^a^	7.82	6.34 ^a^	4.10
COD (mg/L)	26.29 ^a^	6.53	19.74 ^a^	4.02	19.85 ^a^	5.28
TN (mg/L)	2.02 ^a^	0.98	2.03 ^a^	0.18	2.21 ^a^	0.34
TP (mg/L)	0.06 ^b^	0.03	0.12 ^a^	0.07	0.08 ^ab^	0.02
NH_4_^+^-N (mg/L)	0.27 ^a^	0.19	0.55 ^a^	0.27	0.41 ^a^	0.03
NO_3_^−^-N (mg/L)	0.42 ^a^	0.08	0.25 ^a^	0.19	0.25 ^a^	0.26
Chl-a (µg/L)	8.62 ^a^	5.15	17.11 ^a^	27.99	14.60 ^a^	10.99

* T, water temperature; ORP, oxidation–reduction potential; EC, electrical conductivity; DO, dissolved oxygen; Tur, turbidity; COD, chemical oxygen demand; TN, total nitrogen; TP, total phosphorus; NH_4_^+^-N, ammonia nitrogen; NO_3_^−^-N, nitrate nitrogen; Chl-a, chlorophyll-a; s.d., standard deviation. Different letters indicate significant differences.

**Table 2:** Alpha diversity of the bacterial communities in all samples with different groups.

	Richness	Shannon	Ace	Chao1
**June**				
upstream	973 ± 262	4.38 ± 0.21	1730.9 ± 470.9	1446.3 ± 381.6
downstream	673 ± 280	3.37 ± 0.54	1272.9 ± 484.4	1069.4 ± 398.9
**August**				
upstream	685 ± 140	3.86 ± 0.53	1404.0 ± 361.3	1153.1 ± 268.6
downstream	705 ± 135	3.90 ± 0.41	1095.0 ± 154.0	1002.7 ± 173.1
**September**				
upstream	676 ± 127	3.83 ± 0.24	1355.0 ± 350.3	1096.3 ± 251.5
downstream	721 ± 14	4.08 ± 0.19	1170.0 ± 98.3	1043.3 ± 12.0

All values are shown as mean ± s.d.; s.d., standard deviation.

## Supplementary Materials

The following supporting information can be downloaded at: https://www.mdpi.com/article/10.3390/ijerph192013056/s1, Figure S1: Map of the Yongding River showing the sampling sites; Figure S2: Daily average discharge flow from the Guanting Reservoir in 2019; Figure S3: Heatmap showing the microbial community composition at the genus level; Figure S4: The difference in microbial community composition at the genus level; Table S1: Comparison of environmental factors between upstream and downstream subsections in the Beijing Section of the Yongding River; Table S2: Alpha diversity of bacterial communities in all samples; Table S3: Environmental factors influencing microbial communities; Table S4: Correlation between environmental factors.
