# Supplementary material for: Water Quality and Microbial Community in the Context of Ecological Restoration: A Case Study of the Yongding River, Beijing, China
Source: Int J Environ Res Public Health. 2022 Oct 11;19(20):13056. doi: 10.3390/ijerph192013056 (PMC9603554; doi:10.3390/ijerph192013056)
Supplement: Supplementary file 1 [file ijerph-19-13056-s001.zip › ijerph-1881680-supplementary.pdf]

Supplementary Materials:

Water Quality and Microbial Community in the Context of Ecological Restoration: A Case Study of the Yongding River, Beijing, China

Jie Li [1,2], Yujiao Sun [1,*], Xiaoyue Zhang [1], Chengzhong Pan [1], Shurong Zhang [3,*], Binghui Zheng [2]

[1] College of Water Sciences, Beijing Normal University, Beijing 100875, China
[2] National Engineering Laboratory for Lake Pollution Control and Ecological Restoration, Chinese Research Academy of Environmental Sciences, Beijing 100012, China
[3] State Key Laboratory of Earth Surface Processes and Resource Ecology, Faculty of Geographical Science, Beijing Normal University, Beijing 100875, China

* Correspondence: sun201405@163.com (Y.S.); srzhang@bnu.edu.cn (S.Z.)

The Supplementary Materials contain:

- 9 Pages
- 4 Figures
- 4 Tables

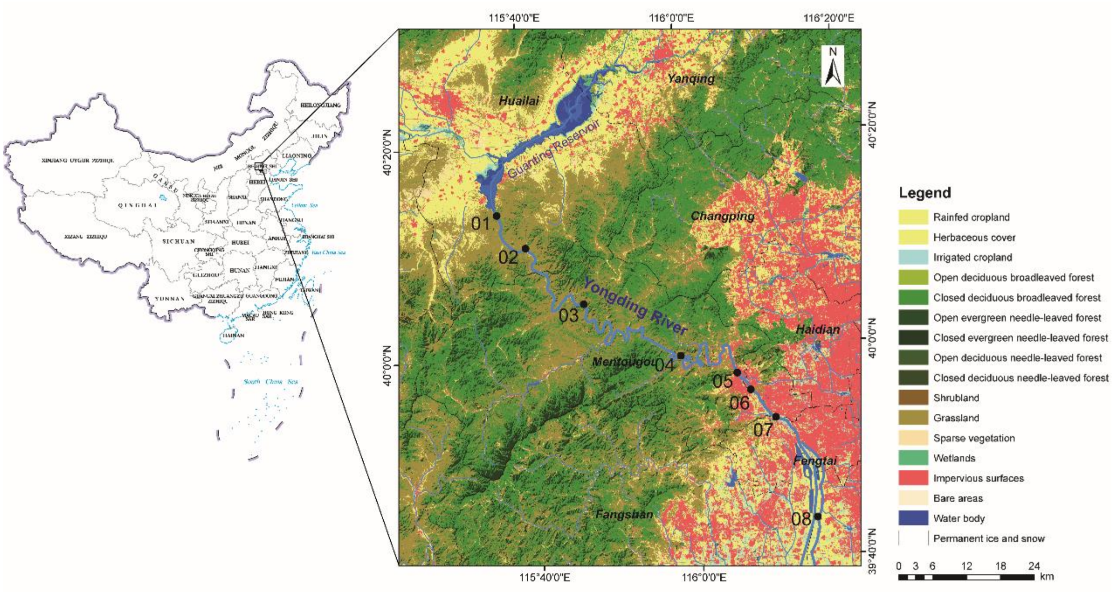

Figure S1. Map of the Yongding River showing the sampling sites. Sites 1-5 are located in the upstream subsection, and sites 6-8 are located in the downstream subsection.

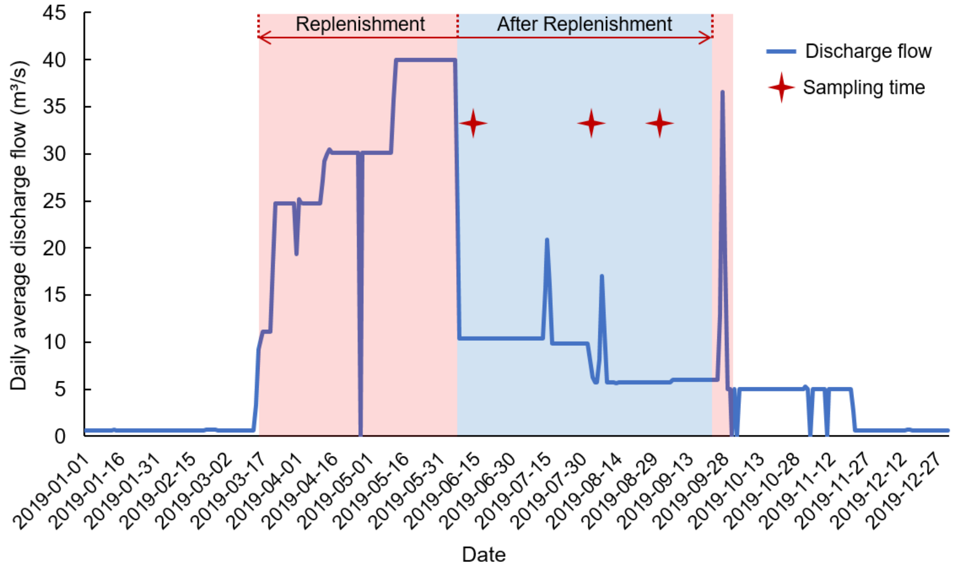

Figure S2. Daily average discharge flow from the Guanting Reservoir in 2019. The region with a red background is defined as the replenishment period, and the region with a blue background is defined as the post water replenishment period. The blue line represents the daily average discharge flow. Red points represent sampling times in June, August, and September.

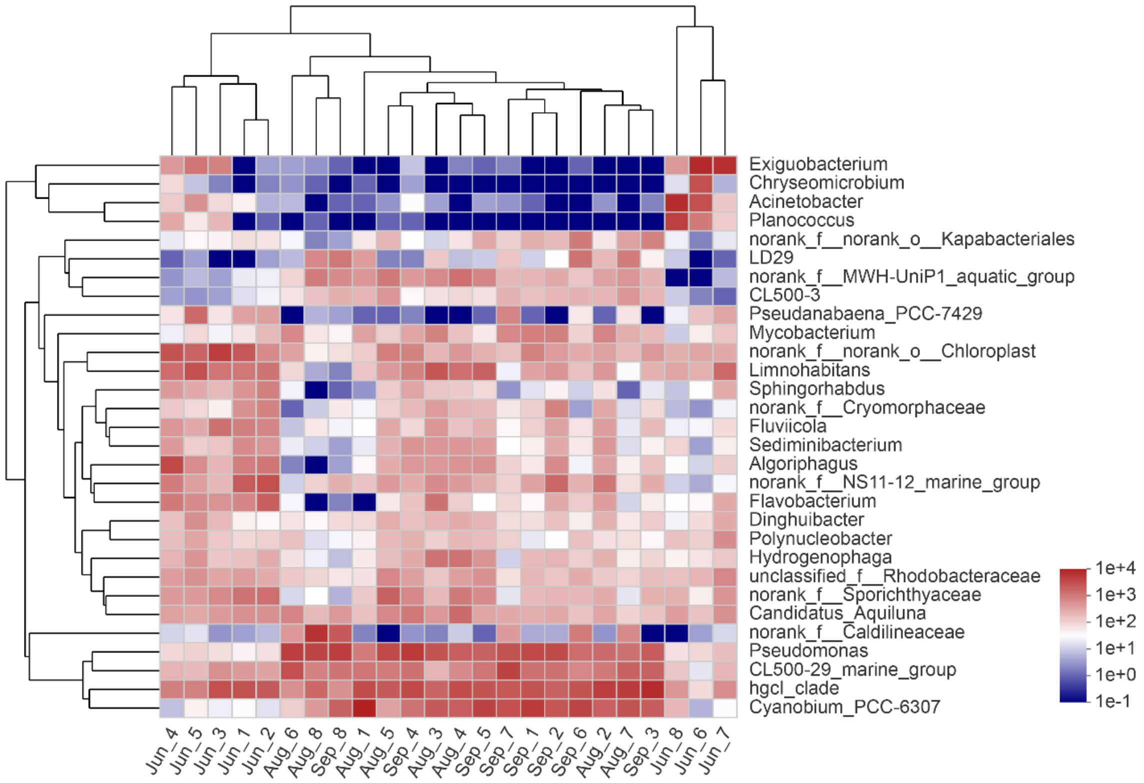

Figure S3. Heatmap showing the microbial community composition at the genus level. Jun, Aug, and Sep represent the sample collection months June, August, and September, respectively.

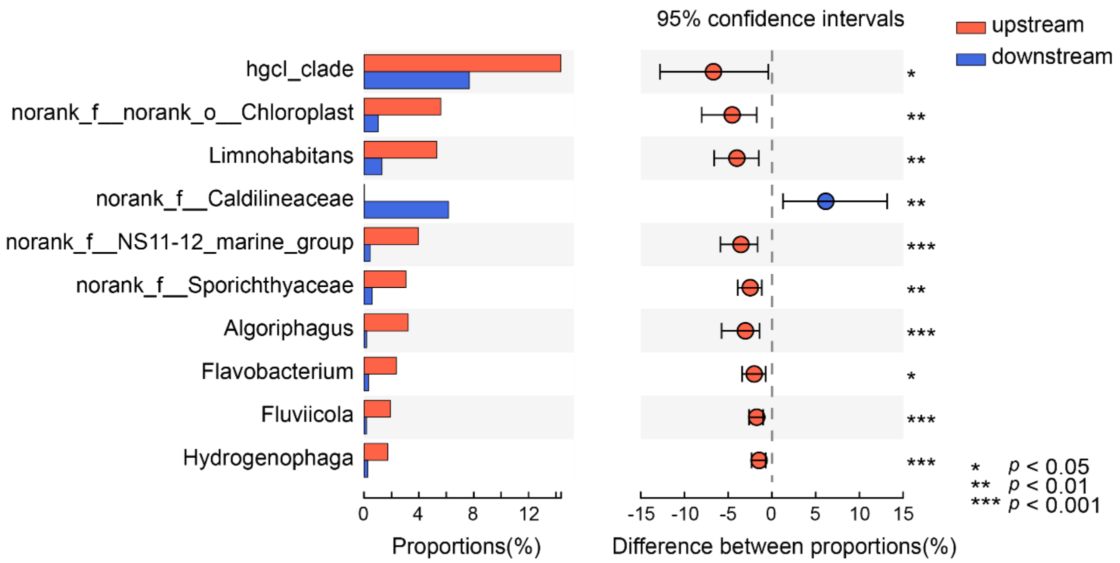

Figure S4. The difference in microbial community composition at the genus level. Upstream and downstream indicate the subsection in which samples were collected.

Table S1. Comparison of environmental factors between upstream and downstream subsections in the Beijing Section of the Yongding River.

Variables *	Upstream		Downstream		p
	Mean	s.d.	Mean	s.d.	
T (°C)	24.73	3.42	27.01	3.45	0.14
pH	8.80	0.15	8.86	0.13	0.40
ORP (mV)	114.49	26.27	112.55	37.77	0.57
EC (μS/cm)	1300.12	452.12	1161.28	143.66	0.65
DO (mg/L)	9.28	1.25	10.88	3.33	0.21
Tur (NTU)	5.30	2.73	10.70	7.53	0.05
**COD (mg/L)**	19.62	5.88	25.86	4.49	**< 0.05**
TN (mg/L)	2.26	0.68	1.80	0.31	0.09
**TP (mg/L)**	0.07	0.03	0.12	0.07	**< 0.05**
NH_4^+-N (mg/L)	0.35	0.14	0.51	0.29	0.23
NO_3^--N (mg/L)	0.36	0.21	0.22	0.16	0.07
**Chl-a (μg/L)**	5.72	3.60	26.31	23.97	**< 0.001**

* T, water temperature; ORP, oxidation–reduction potential; EC, electrical conductivity; DO, dissolved oxygen; Tur, turbidity; COD, chemical oxygen demand; TN, total nitrogen; TP, total phosphorus; NH_4^+-N, ammonia nitrogen; NO_3^--N, nitrate nitrogen; Chl-a, chlorophyll-a; s.d., standard deviation; *p*, level of significance based on the Mann–Whitney U test.

Table S2. Alpha diversity of bacterial communities in all samples.

Samples	Richness	Shannon	Ace	Chao1
Jun_1	528	4.11	1047.5	775.6
Jun_2	830	4.22	1286.2	1278.5
Jun_3	1085	4.37	1992.6	1623.3
Jun_4	1191	4.48	2167.0	1781.2
Jun_5	1229	4.71	2161.2	1772.9
Jun_6	550	3.04	1262.8	1046.4
Jun_7	1060	4.14	1871.1	1569.1
Jun_8	409	2.94	684.7	592.8
Aug_1	465	2.83	788.1	681.6
Aug_2	885	4.27	1842.0	1513.9
Aug_3	758	3.96	1614.9	1239.4
Aug_4	698	4.02	1522.6	1180.1
Aug_5	618	4.22	1252.5	1150.3
Aug_6	891	4.27	1260.0	1246.5
Aug_7	653	4.09	889.3	899.6
Aug_8	572	3.33	1135.8	862.0
Sep_1	665	3.61	1138.2	959.4
Sep_2	918	4.20	2001.4	1588.8
Sep_3	637	3.63	1011.9	930.2
Sep_4	546	3.66	1195.3	932.3
Sep_5	612	4.04	1428.3	1070.7
Sep_6	741	4.33	1189.8	1059.8
Sep_7	713	4.03	1279.3	1038.6
Sep_8	710	3.89	1041.0	1031.4

Table S3. Environmental factors influencing microbial communities.

	**CCA1**	**CCA2**	**R^2**	***p***
**T**	-0.728	-0.686	0.440	**0.001**
**pH**	-0.833	-0.553	0.511	**0.002**
ORP	0.691	0.723	0.023	0.774
EC	-0.109	0.994	0.164	0.165
DO	-0.665	-0.747	0.210	0.084
Tur	-0.659	-0.752	0.138	0.182
COD	0.979	-0.203	0.128	0.213
**TN**	-0.655	0.756	0.293	**0.031**
**TP**	-0.449	-0.894	0.347	**0.029**
**NH_4^+-N**	-0.345	-0.939	0.308	**0.038**
**NO_3^--N**	0.717	0.698	0.397	**0.004**
Chl-a	-0.309	-0.951	0.200	0.084

T, water temperature; ORP, oxidation–reduction potential; EC, electrical conductivity; DO, dissolved oxygen; Tur, turbidity; COD, chemical oxygen demand; TN, total nitrogen; TP, total phosphorus; NH_4^+-N, ammonia nitrogen; NO_3^--N, nitrate nitrogen; Chl-a, chlorophyll-a.

Table S4. Correlation between environmental factors.

	T	pH	ORP	EC	DO	Tur	COD	TN	TP	NH_4^+-N	NO_3^--N	Chl-a
T	1.000											
pH	**0.749****	1.000										
ORP	**0.477***	0.257	1.000									
EC	0.210	0.339	**0.478***	1.000								
DO	**0.500***	0.371	0.197	-0.004	1.000							
Tur	0.336	0.281	0.115	-0.241	0.053	1.000						
COD	-0.186	-0.030	0.148	0.239	-0.094	0.094	1.000					
TN	0.147	0.161	-0.083	0.284	0.295	-0.397	-0.288	1.000				
TP	**0.614****	**0.560****	0.199	0.149	0.237	0.402	0.049	-0.118	1.000			
NH_4^+-N	**0.586****	0.351	0.173	-0.376	**0.413***	**0.428***	**-0.460***	-0.041	0.357	1.000		
NO_3^--N	-0.402	**-0.507***	0.093	-0.235	-0.271	-0.281	-0.202	-0.092	**-0.590****	-0.149	1.000	
Chl-a	0.107	0.088	-0.141	-0.190	0.146	0.403	0.373	-0.345	**0.576****	0.164	**-0.562****	1.000

T, water temperature; ORP, oxidation–reduction potential; EC, electrical conductivity; DO, dissolved oxygen; Tur, turbidity; COD, chemical oxygen demand; TN, total nitrogen; TP, total phosphorus; NH_4^+-N, ammonia nitrogen; NO_3^--N, nitrate nitrogen; Chl-a, chlorophyll-a. *p*, level of significance based on Spearman's correlation. *, $p < 0.05$; **, $p < 0.01$.
